# Permutation Statistics for Connectivity Analysis between Regions of Interest in EEG and MEG Data

**DOI:** 10.1038/s41598-019-44403-z

**Published:** 2019-05-28

**Authors:** Fahimeh Mamashli, Matti Hämäläinen, Jyrki Ahveninen, Tal Kenet, Sheraz Khan

**Affiliations:** 1 Department of Neurology, MGH, Harvard Medical School, Boston, MA USA; 2 Athinoula A. Martinos Center for Biomedical Imaging, MGH/HST, Boston, MA USA; 3 Department of Radiology, MGH, Harvard Medical School, Boston, MA USA; 4 McGovern Institute for Brain Research, Massachusetts Institute of Technology, Cambridge, MA USA

**Keywords:** Statistical methods, Neuroscience, Outcomes research

## Abstract

Connectivity estimates based on electroencephalography (EEG) and magnetoencephalography (MEG) are unique in their ability to provide neurophysiologically meaningful spectral and temporal information non-invasively. This multi-dimensional aspect of the MEG/EEG based connectivity increases the challenges of the analysis and interpretation of the data. Many MEG/EEG studies address this complexity by using a hypothesis-driven approach, which focuses on particular regions of interest (ROI). However, if an effect is distributed unevenly over a large ROI and variable across subjects, it may not be detectable using conventional methods. Here, we propose a novel approach, which enhances the statistical power for weak and spatially discontinuous effects. This results in the ability to identify statistically significant connectivity patterns with spectral, temporal, and spatial specificity while correcting for multiple comparisons using nonparametric permutation methods. We call this new approach the *P*ermutation *S*tatistics for *C*onnectivity *A*nalysis between *R*OI (PeSCAR). We demonstrate the processing steps with simulated and real human data. The open-source Matlab code implementing PeSCAR are provided online.

## Introduction

EEG and MEG are ideal techniques to non-invasively measure brain activity with high temporal-spectral and reasonable spatial resolution^1–6^. Typically, neuroscience studies relate particular variation in the brain such as source activation, inter-regional functional connectivity, or oscillatory power to certain experimental paradigm or behavioral measures. However, within this multifaceted data, finding the modulation associated with a particular effect or contrast of interest in the brain can be challenging. This challenge is particularly pronounced in functional connectivity analysis. Depending on the hypothesis and the experiment, the functional connectivity is assessed either from a seed region to the rest of the brain^7–9^ or between all atlas-based (cortical) parcels^10–13^. In the former, cluster-based statistics is used to find differences between experimental conditions in time, frequency, and space^9,14,15^. However, this approach provides sufficient statistical power for detecting differences across the experimental manipulations of interest only when the effect sizes are very large and the clusters are spatially continuous across the cerebral cortex. In all-to-all parcel comparisons, a simplified strategy is often taken by averaging the functional connectivity estimates within certain time and frequency bands such as alpha (8–12 Hz) or beta (13–30 Hz), but this results in the loss of temporal and spectral specificity. In both of the aforementioned approaches, one has to correct for a massive number of comparisons in order to control the family-wise error rates (FWER). In addition, searching within the whole brain may, on the one hand, increase the risk of false positives and, on the other hand, reduce the sensitivity for weaker but functionally relevant connections.

**Figure 1:**
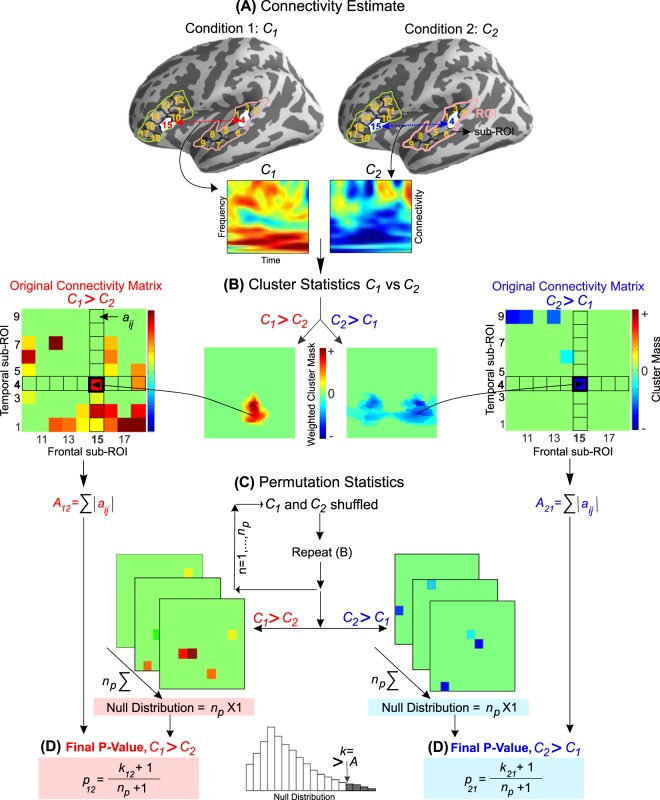
Schematic representation of PeSCAR: (**A**) ROIs in left STG and IFG based on freesurfer parcellation with 9 sub-ROIs within each. For both conditions (C_1_ and C_2_) the connectivity is estimated as a function of time and frequency for all sub-ROI pairs; an example for the sub-ROI pair (4, 15) is shown. (**B**) Cluster statistics is computed between C_1_ and C_2_ in time and frequency between all sub-ROI pairs. The example shows the cluster mask weighted by t-values for both C_1_ > C_2_ and C_2_ > C_1_. for the for sub-ROI pair (4, 15) pair. The sum over all frequencies and times of the weighted cluster mask is called the cluster mass. Original connectivity matrix is generated for each comparison in which each element will be either zero (p > 𝛼) or the cluster mass (p < 𝛼), where 𝛼 is the chosen threshold. Each element of original connectivity matrix is referred to as a_ij_ and the sum across all sub-region pairs is called “original total connectivity” A. (**C**) C_1_ and C_2_ are pooled and shuffled n_p_ times. Cluster statistics (**B**) are then recom_p_uted for each shuffle and the null distribution is generated. (**D**) The p-value for each comparison (C_1_ > C_2_ and C_2_ > C_1_) is computed according to the formula shown, where k_12_ and k_21_ is the number of times that the null distribution is larger than the A_12_ or A_21_, respectively.

An alternative approach is to focus on particular regions in the brain based on prior hypothesis. In functional connectivity analysis, such ROIs are either selected with help of source localization or manually delineated for each individual based on specific criteria, such as task dependent modulation in activity; e.g.^16–24^. In conventional MEG or EEG studies, these ROIs also need to be anatomically small in order to reduce temporal signal cancellations. This approach relies on a robust estimate of activity in the ROIs and may not be applicable when weak cognitive effects are investigated. In many situations the ROIs are selected using existing anatomically and functionally defined parcellations. However, these parcellations are agnostic to variations of the effects of interest within the ROI. That means that effect can be sometimes be missed when averaging across an entire ROI. For an extreme example, in a motor task, moving the hand and the foot does not activate the entire motor cortex but specific parts of it according to the motor homunculus. If one averages over the entire motor cortex, signal to noise ratio will be reduced and physiologically important spatial specificity is lost. Therefore, there is an advantage of sub-dividing an ROI into smaller pieces, which we call sub-ROIs. If there is an effect within the ROI, the sub-ROIs should show the same effect. Particularly for discontinuous effects, sub-division of ROIs would increase the spatial specificity and statistical power. Notably, inherent discontinuities are also caused by electric/magnetic field cancellations when the activations span across the opposite banks of cortical gyri and sulci^25^. Examples of ROIs and sub-ROIs is in Fig. 1A.

In this paper, we propose a novel approach to conduct ROI-based connectivity analysis. The ROI can be as large as existing functionally or anatomically defined parcellations, thus can be purely chosen by prior hypothesis. To correct for multiple comparisons across the sub-ROIs, we employed a non-parametric permutation method^26^, which has been successfully applied before^15,27–36^. We name this method PeSCAR, which stands for *P*ermutation *S*tatistics for *C*onnectivity *A*nalysis between *R*OI. The PeSCAR algorithm will automatically select the functionally relevant sub-ROIs, hence enhancing spatial specificity. In contrast to cluster statistics, this approach increases the statistical power for weak and spatially discontinuous effects while maximizing spectral and temporal specificity. The application of our approach is not limited to connectivity; it can be employed in simpler scenarios such as (A) comparing source activations within ROI, (B) inter-measure correlations, e.g. correlation between ROI activation and behavioral measurements and (C) comparing time-frequency maps within an ROI, see Supplementary Information (SI). We demonstrate PeSCAR in the case of functional connectivity, as this is the most complex analysis among the aforementioned ones. Using simulation and real data set we show the processing steps.

## Results

### The PeSCAR method

In MEG-EEG studies, the statistical comparison is typically between conditions across a group of subjects or between groups of people conducting the same experiment. If the activation inside each ROI is distributed uniformly across the entire ROI, one could simply average the activity inside the ROI and conduct the functional connectivity analysis on the averaged time series. However, in the majority of MEG and EEG studies, this may not be the case due to the different spatial sensitivity of MEG and EEG to sulci and gyri. This problem becomes more challenging because of the poor signal-to-noise ratio typically encountered in conventional MEG and EEG studies. Therefore, the estimated activation inside an ROI can consist of discontinuous patches. In addition, in realistic data, there is variability across the subjects. Hence, traditional spatial averaging might result in removal or reduction of the information that is present at lower aggregation. Moreover, this type of activation is less likely to be found by cluster statistics, which prefers large anatomically continuous activations. To overcome these limitations, PeSCAR divides each ROI into sub-ROIs and computes connectivity between all possible sub-ROI pairs.

We have incorporated these ideas to the PeSCAR pipeline, illustrated in Fig. 1. Here, for simplicity, we illustrate a particular case and give a generalization in SI. Let us assume there is one group of participants in an experiment with two conditions. The goal is to compare the functional connectivity between ROI_1_ and ROI_2_ between two conditions (*C*_1_ and *C*_2_) across all subjects. The functional connectivity is estimated in time and frequency: *t*_0_ < *t *<*t*_1_ and *f*_0_ < *f* <*f*_1_, respectively. The null hypothesis is that functional connectivity is not different between *C*_1_ and *C*_2_. It has been shown that this is equivalent to the exchangeability hypothesis, i.e., *C*_1_ and *C*_2_ are exchangeable (see statistical proof in Maris, Oostenveld, 2007). The resulting analysis steps are:

(i) Divide each ROI into sub-ROIs of approximately equal size. This leads to *N* and *M* sub-ROIs for ROI_1_ and ROI_2_, respectively (Fig. 1A).
(ii) Estimate the functional connectivity between the sub-ROIs in time and frequency for both conditions and all subjects (Fig. 1A). This leads to an *N* × *M* matrix of connectivity values for each subject and condition.
(iii) Compute cluster statistics in time and frequency between *C*_1_ and *C*_2_ each sub-ROI pair^33^. Here, the appropriate test statistic is the paired *t*-test. It is important to note that the cluster statistics provide a *p*-value, cluster mass, cluster mask and the *t*-values of the time-frequency plane (see Fig. 1 for an illustration). The *p*-value shows whether the cluster is significant after correction for multiple comparisons in time and frequency. The cluster mass is the sum of the *t*-values of every time-frequency pair inside the cluster. Positive and negative cluster masses indicate *C*_1_ > *C*_2_ and *C*_2_ > *C*_1_, respectively. The cluster mask is a binary matrix covering the time-frequency plane; it indicates whether a particular time-frequency pair belongs to the cluster (Fig. 1B).
(iv) Generate two *N* × *M* matrices, one for the *C*_1_ > *C*_2_ and the second for *C*_2_ > *C*_1_. Each element is set to zero if *p* > *α*, where *α* is the chosen threshold, for that particular sub-ROI_1_-sub-ROI_2_ pair. If *p* < *α*, then the element of the matrix is set to the cluster mass. According to Maris and Oostenveld, 2007, “this threshold may or may not be based on the sampling distribution of the t -value under the null hypothesis, but this does not affect the validity of the nonparametric test”. In our simulations, the threshold α was the 97.5^th^ percentile point of a T-distribution, which is used as a critical value in a parametric two-sided t -test at alpha-level 0.05. We call these thresholded cluster mass values, forming an *N *× *M*, *original connectivity matrix* (Fig. 1B) with each element as *a*_*ij*_. We then sum the elements of each original connectivity matrix (*a*_*ij*_) resulting in the *original total connectivity*: $A={\sum }_{j=1}^{M}{\sum }_{i=1}^{N}|{a}_{ij}|,$for the two comparisons *C*_1_ > *C*_2_ and *C*_2_ > *C*_1_, as ${A}_{12},\,{A}_{21}$ respectively. PeSCAR can be easily extended to employ a threshold-free version of cluster statistics to avoid the problem of threshold selection^37^.
(v) We then permute the two conditions *n*_*p*_ times (*n*_*p*_ > 1000) and repeat steps (iii) and (iv) for each permutation. After going through all permutations, we will end up with two *n*_*p*_ arrays of values, which we will use to derive the null distributions for *C*_1_ > *C*_2_ and *C*_2_ > *C*_1_ (Fig. 1C). Finally, we compare the ${A}_{12}\,{\rm{and}}\,{A}_{21}$ from step (iv) to the empirical null distribution from step (v) to assign $p$-values for *C*_1_ > *C*_2_ and *C*_2_ > *C*_1_ respectively (Fig. 1D). The *p*-values will be computed as ${p}_{12}=({k}_{12}+1)/({n}_{p}+1)$ and ${p}_{21}=({k}_{21}+1)/({n}_{p}+1)$, where *k*_12_ and *k*_21_ are the numbers of times the null distribution is larger than the *A*_12_ and A_21_ for *C*_1_ > *C*_2_ and *C*_2_ > *C*_1_, respectively. In order to correct for the two comparisons, we apply Bonferroni correction to both *p*_12_ and *p*_21_.

There are two approaches to visualize the time-frequency map of the difference between *C*_1_and *C*_2_, un-weighted and weighted. In the un-weighted case, the binary cluster masks for all significant detections from step (iv) are summed up. Thus, this quantity indicates how many significant connections are present for each time-frequency pair. In the weighted case, the binary cluster masks are weighted by the *t*-values from step (iii) before summation according to the significance.

Using simulations, we demonstrate that when the SNR is low, PeSCAR offers more statistical power than alternative conventional averaging approach, in which time series is averaged over vertices across ROI and the connectivity is estimated on the averaged time series and the cluster statistics in time and frequency is used for contrast. To compare the two approaches, we estimated the statistical sensitivity or power by generating 100 data sets under the alternative hypothesis. In this simulation, we randomly draw different effect configurations and add a fraction of resting state noise. The resting state noise ratio was drawn from a uniform distribution. This means that both strong and weak effects are considered in this simulation. The proportion of data sets for which the null hypothesis is rejected, is the sensitivity or statistical power.

### Spatially continuous (non-scattered) sources: varying SNR

**Figure 2:**
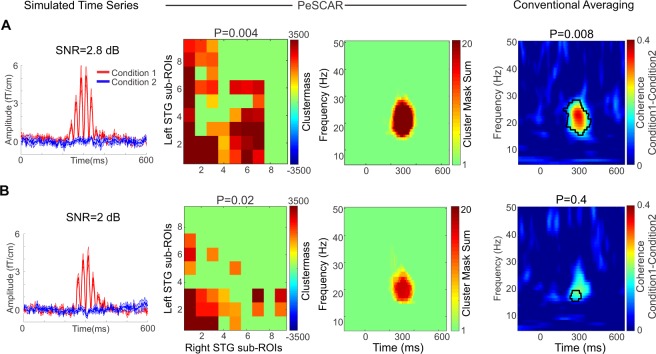
Left column: Average simulated time series across eight subjects in sensor space for three tested SNR (**A**,**B**). Middle columns: PeSCAR results comparing Left-right STG functional connectivity between two simulated conditions for three SNR of 2.8 and 2 (**A**,**B**). Matrix of sub-ROI pairs that reached significance (original total connectivity) and the un-weighted time frequency map of the difference between two conditions are demonstrated. Right column: Average time-frequency map of the coherence difference between the two conditions and the cluster statistics results in three SNR (**A**,**B**).

**Figure 3:**
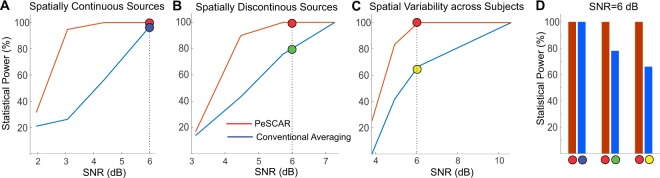
Statistical power of PeSCAR and conventional averaging approach for three scenarios. (**A**) Spatially continuous or non-scattered sources. (**B**) Spatially discontinuous or scattered sources and (**C**) Variable and discontinuous sources across subjects. (**D**) Statistical power for SNR = 6 dB for all scenarios. Colored circles shows the relevant point in (**A**–**C**).

We considered two ROIs in left and right STG and each ROI were divided into 9 equal size sub-ROIs. Two conditions were simulated (*C*_1_ and *C*_2_). For all subjects and *C*_1_, 50 epochs were simulated in the time range of −250 to 750 ms and frequency range of 15 to 20 Hz within the first three sub-ROIs in left and right STG. The signal was limited for the time between 200 and 400 ms in time. As *C*_2_, 50 epochs were randomly selected from artifact free resting state data. The coherence between all sub-ROI pairs was estimated and PeSCAR was then calculated. We varied the SNR in *C*_1_, which would make detecting the difference between two conditions harder. As examples, we demonstrate the results for two different SNRs. The average time series across subjects in sensor space for each condition and the corresponding SNR is displayed in Fig. 2 (column 1). Using PeSCAR, the simulated difference between the conditions (*C*_1_ > *C*_2_) was revealed with P-values of 0.004 and 0.02 for SNR values of 2.8 and 2 dB, respectively (Fig. 2A,B – columns 2–3). Using conventional averaging approach, results similar to PeSCAR were found for SNR of 2.8 dB. However, when the SNR was reduced to 2 dB, the alternative method failed in finding the simulated difference between the two conditions. In Fig. 3A, we show the statistical power as a function of SNR (Fig. 3A). At high SNR, both methods have 100% sensitivity, while at lower SNRs, PeSCAR detects the effect with higher sensitivity

### Spatially discontinuous (scattered) sources

**Figure 4:**
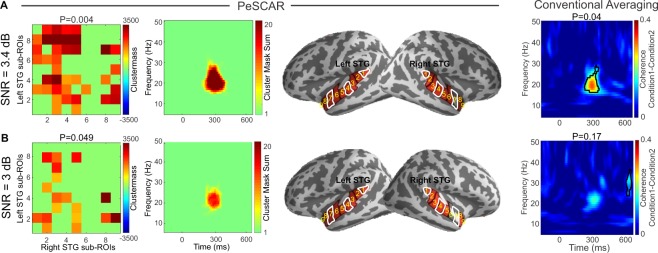
Left-right STG functional connectivity comparison when the sources are discontinuous across surface tested for two SNRs. The actual simulated signal was placed in the sub-ROIs with white color border. In each panel, from left, first column (**A**,**B**) is the original total connectivity matrix of sub-ROI pairs that reached significance, second column (**A**,**B**) is the un-weighted time frequency map of the difference between conditions, third column (**A**,**B**) is the sub-ROIs on the cortex and fourth column (**A**,**B**) is the average time-frequency map of the coherence difference between two conditions with cluster statistics results demonstrated.

To simulate spatially discontinuous sources, the activation was placed inside the sub-ROIs 1, 4, 7, marked with white borders in (Fig. 4-third column). We show two SNRs (3.4 and 3 dB) as examples. In the case of 3.4 dB, PeSCAR and conventional averaging approach found similar differences between the two conditions: PeSCAR with a P = 0.004 and the conventional averaging with a P = 0.04, see Fig. 4. At the lower SNR of 3 dB, shown in Fig. 4B, only PeSCAR could find the difference between two conditions. In Fig. 3B, we show the statistical power for various SNR comparing both approaches. Note that in case of spatially discontinuous sources, we need a higher SNR to detect an effect to be significant than when the sources are spatially continuous. We marked SNR = 6 dB in panels A and B of Fig. 3 to show that when the sources are discontinuous, PeSCAR still offers 100% sensitivity while the conventional averaging sensitivity reduces to 78% (Fig. 3D).

### Spatially discontinuous (scattered) sources: spatial variability across subjects

**Figure 5:**
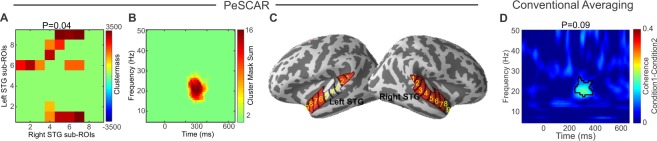
Functional connectivity differences between two conditions when the simulated signal varied spatially across subjects. (**A**) The original total connectivity matrix of sub-ROI pairs that reached significance, (**B**) the un-weighted time frequency map of the difference between conditions and (**C**) is the sub-ROIs on the cortex. (**D**) Average time-frequency map of the coherence difference between two conditions with cluster statistics results demonstrated.

To simulate a more realistic scenario, we introduced spatial variability across subjects by activating different sub-ROI sets in different subjects. Three sub-ROIs in each ROI were randomly activated across subjects, with additional constraint that in the right STG the sources are spatially discontinuous, which is the most challenging scenario. For example, when PeSCAR found the difference between the two conditions with a P = 0.04, conventional averaging resulted in P = 0.09 (Fig. 5). The statistical power for PeSCAR and conventional averaging is shown Fig. 3C, which indicates that with variability across subjects, PeSCAR is more robust. We also see that a higher SNR is required in this scenario to find an effect between the two conditions. Interestingly, with SNR = 6 dB, PeSCAR still offers 100% sensitivity, whereas with conventional averaging sensitivity reduces to 65% (Fig. 3D).

### PeSCAR application to human data: auditory mismatch paradigm

**Figure 6:**
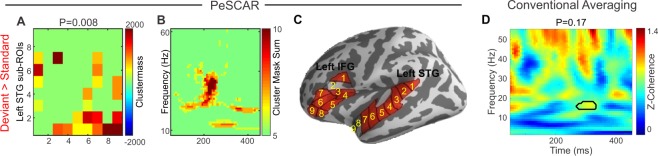
Left temporal-frontal functional connectivity comparison between deviant and standard conditions from a real human dataset. (**A**) The original total connectivity matrix of sub-ROI pairs that reached significance. (**B**) Un-weighted time-frequency (TF) map of the difference between standard and deviant. (**C**) Sub-ROIs on the cortex. (**D**) Average time-frequency map of the coherence difference between two conditions with conventional cluster statistics results is demonstrated.

MEG data from an auditory mismatch paradigm consisting of standard and deviant tones were used^20^. STG and inferior frontal gyrus (IFG) have been consistently found to underlie auditory mismatch responses. Therefore, left STG and left IFG were considered as ROIs and the coherence between them estimated in time and frequency. In addition, to reduce the point spread effect we normalized the coherence of deviant vs standard following^33^. Using PeSCAR, stronger left temporal-frontal connection for deviant than standard were found around 200 ms and between 20–40 Hz with a p-value of 0.008 (Fig. 6). Original total connectivity matrix and un-weighted time-frequency map of the connectivity difference between standard and deviant is shown in (Fig. 6A,B). Those sub-ROIs in left STG and left IFG that found to show condition dependent effect are marked in Fig. 6C. Our findings are consistent with a previous study, which showed 4–25 Hz left temporal frontal functional connectivity for deviant in contrast to standard tones using sensor space data analysis^38^. PeSCAR in contrast offers conducting statistics in the source space with higher spectro-temporal resolution. Lack of a standard method in the field to perform ROI based functional connectivity analysis has limited the studies to pursue source-based connectivity analysis in time and frequency. The conventional within-ROI averaging approach did not show any significant differences between the conditions (Fig. 6D).

## Discussion

With increasing interest in the connectivity analysis in general and in the MEG/EEG domain in particular, there is a growing need to develop statistical approaches that identify functionally relevant connections with increased spatial, temporal, and spectral specificity. Unfortunately, a generally accepted method for ROI-based functional connectivity analysis of MEG/EEG data has so far been lacking, and previous studies have therefore been limited to using tailored heuristic approaches. Here, we present a novel approach to conduct statistics for ROI based functional connectivity analysis in MEG/EEG data. This PeSCAR method could offer substantially greater power if the effect of interest is spatially distributed, particularly when the contrast to noise ratio could be low.

We assessed the performance of PeSCAR using both multiple simulations and a real data set from human recordings. The simulation results show that when the SNR is high, conducting functional connectivity analysis on the averaged time series across entire ROI offers enough statistical power to detect the effect between the conditions. However, our simulation results also demonstrated that in cases when the SNR is poor, the novel PeSCAR method offers more statistical power than the conventional averaging method. Our simulation results were, finally, verified by using connectivity analyses of a real data set, obtained from an auditory “mismatch negativity” experiment in humans. Whereas the conventional analysis of these real data showed only weak connectivity effects at the low beta band, the PeSCAR analyses demonstrated robust connectivity differences between automatic auditory change detection vs. standard-sound processing conditions, which were spread into various time and frequency bands. In the cognitive neuroscience sense, this would mean that instead of a local auditory cortex process^39,40^, the detection of unexpected changes in the auditory stream involves a broader frontotemporal network (i.e., “predictive coding”)^41^. This result provides a notable practical example how a high-resolution time-frequency map of the effect of interest provided by PeSCAR can have a major theoretical impact the interpretation of the results.

We should note that PeSCAR might face computational limitations when multiple statistical contrasts are compared such as ANOVA. However, with the advent of graphics processing units (GPU), this problem can be addressed. It is also worth noting that similarly to other permutation or randomized simulation statistics methods to address FWE, PeSCAR can only be used only when the assumption of exchangeability holds. These notions need to be considered before conducting connectivity analyses using PeSCAR.

In summary, there are now four approaches to conduct functional connectivity analysis for MEG/EEG data: (1) from a seed region to the rest of the brain (2) between ‘all-to-all’ atlas-based parcellations (3) conventional averaging, and 4) PeSCAR. Each has its own advantages and disadvantages, which have been discussed in this paper. If the SNR is high and the activations are genuinely continuous across the cortex, generic procedures that operate on the all-to-all ROI connectivity comparisons or cluster-based statistics in the whole cortex can presumably provide enough power to declare the effect of interest significant. However, we believe that PeSCAR will play an important role in neuroscience studies by offering a novel way to detect functionally relevant patterns, hidden in the multifaceted MEG/EEG data.

## Methods

All experimental protocols were approved by the Massachusetts General Hospital institutional review board. Participants were consented in accordance with the approved protocol and all methods were carried out in accordance with relevant guidelines and regulations.

### Simulations

All simulations were undertaken using a whole-head VectorView MEG system (Elekta-Neuromag), comprised of 306 sensors arranged in 102 triplets of two orthogonal planar gradiometers and one magnetometer. Eight healthy subjects’ data were used who has previously participated in a resting state experiment. The location of the brain anatomy with respect to the sensors was taken for each subject. The sampling frequency of the data was 600 Hz. MEG resting state data were spatially filtered using the signal space separation method (Elekta-Neuromag Maxfilter software) to suppress noise generated by sources outside the brain^42,43^. Cardiac and ocular artifacts were removed by signal space projection^1^. The data were filtered between 0.1 and 140 Hz. Resting state data of each subject was used as the biological noise that was added to the simulated data in a later stage. In contrast to the empty room recording, resting state noise data is a more realistic noise because it takes into account the specific covariance structure between brain regions. The MEG forward solution was computed using a single-compartment boundary-element model (BEM) assuming the shape of the intracranial space^44^. The current distribution was estimated using the regularized L2 minimum-norm estimate (MNE) with the regularization parameter set to 0.1. The source orientations were fixed to be perpendicular to the cortex. The noise covariance matrix that was used to calculate the inverse operator was estimated from data acquired before each session in the absence of a subject. The activity inside each ROI was simulated for two conditions and eight subjects in order to allow us to do the permutation statistics. We assess the validity of the method using several simulations and comparing with conventional existing methods as following.

#### Spatially continuous sources: Varying signal to noise ratio

Two ROIs in left and right superior temporal gyrus (STG) were selected according to Freesurfer parcellation^45^. Each ROI was divided into 9 sub-ROIs using an automatic routine offered in the Freesurfer suite. All sub-ROIs in both ROIs were of approximately the same size. For all subjects and condition one, 50 epochs were simulated in the time range of −250 to 750 ms and frequency range of 15 to 20 Hz. The activity was generated in the first three sub-ROIs of left STG and right STG using the following equation:

1 $$X(v,e,t)={q}_{0}\frac{1}{F}\sum _{f\in \Omega }(w(t)\ast \sin (2\pi ft))$$

**Figure 7:**
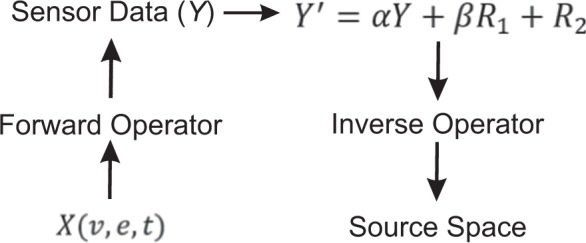
A schematic that illustrates the steps to generate the simulated signal in one sub-ROI. Amplitude of Y is reduced using *α*, which is selected between 0.04 and 0.1. *β* is a number drawn from uniform distribution and *R*_1_ and *R*_2_ represent two separate segments of resting state data.

*X* represents a three-dimensional matrix [Vertices (*v*) × Epochs (*e*) × Time (*t*)]. In this equation, *w(t)* is the Hann window $(w(t)=\frac{1}{2}(1-\,\cos (\frac{2\pi t}{T}))$, *t* is the time, *f* is the frequency in the set $\Omega =\{15,\ldots ,20\}$ ${\rm{Hz}}$, and *F* = 6 is the number of frequencies in Ω. The signal was confined to *t* = 200 and 400 ms and the rest of the data were set to zero. This would allow us to expect a particular activation in time and frequency. The overall amplitude of the currents was set to *q*_0_ = 10 nAm/mm^2^ as suggested by a previous study^46^. The corresponding sensor-space signals Y were obtained by multiplying the source space signals by the forward operator. We used a scale *α* to signal Y to reduce the SNR and make it more comparable to real data (Fig. 7). The value of *α* was randomly selected between 0.04 to 0.1. The resting-state data of each subject was added to this signal by selecting a 1000 ms segment *R*_2_ randomly from 7 minutes of resting-state data recorded in each subject, see Fig. 7. Segments of the resting state data which had a peak-to-peak amplitude larger than 1000 fT/cm and 3000 fT in any of gradiometers and magnetometers, respectively, were not considered. To perform power analysis and generate 100 data sets, we additionally added a fraction of another segment of resting state noise to vary the SNR, represented with *R*_1_ in Fig. 7. The amplitude of *R*_1_ was varied with *β* value drawn from a uniform distribution between 0 and 1. The SNR of *Y*′ was computed as follows:

2 $$20\times {\mathrm{log}}_{10}\frac{S}{N}$$

here, S is the average of the absolute value of the signal across all sensors within the time interval of 200–400 ms of condition 1. N is the average of the absolute value of the resting state noise (condition 2) within all time intervals across all sensors. As condition two, only resting state data were used for each subject. Fifty epochs were randomly selected from 7 minute of each subject that had no overlaps with those in condition one. Both conditions data in the sensor space was projected to the source space using the inverse operator. All sub-ROIs time series were extracted (see SI) and the coherence between all sub-ROI pairs were estimated (see SI). PeSCAR was then applied to this simulated data.

#### Spatially discontinuous sources

To simulate spatially discontinuous sources, the signal from previous section was placed within those sub-ROIs that had spatial distance of two sub-ROIs. Thus, signal was generated within sub-ROI number 1, 4, 7 in both left and right STG. In Fig. 4, the location of sub-ROIs in left and right STG are marked with white color borders. All the other steps remain like previous section.

#### Spatially discontinuous sources: Spatial variability across subjects

In realistic data, source activity or localization may be spatially discontinuous and also vary across subjects. To test this effect, we repeated our simulation, with the exception that those 3 sub-ROIs that have signal were randomly selected among 9 sub-ROIs across all subjects in left STG. In right STG, for each subject, those 3 sub-ROIs with the signal were randomized across subjects and were spatially discontinuous also. For example, in subject 1, in right STG, sub-ROI of 1, 3, and 5 had activation, in subject 2, sub-ROI of 2, 4, 6 had activation and etc. This introduces variability across all subjects. In addition, SNR was reduced using the scaling factor of 0.06.

#### Simulation scripts

All of the simulation scripts in Matlab are available online via our github repository (https://github.com/FahimehMamashli/PeSCAR). In future, Python implementation will be available as well.

## Supplementary information

supplementary info

## Data Availability

All data will be made available upon request.
