# Supplementary material for: Permutation Statistics for Connectivity Analysis between Regions of Interest in EEG and MEG Data
Source: Sci Rep. 2019 May 28;9:7942. doi: 10.1038/s41598-019-44403-z (PMC6538606; doi:10.1038/s41598-019-44403-z)
Supplement: Supplementary file 1 — supplementary info [file 41598_2019_44403_MOESM1_ESM.pdf]

Supplementary Information

Permutation Statistics for Connectivity Analysis between Regions of Interest in EEG and MEG Data

Fahimeh Mamashli, Matti Hämäläinen, Jyrki Ahveninen, Tal Kenet*, Sheraz Khan*

**Equal Contribution*

Generalizability of the approach

This method can be generalized to other statistical contrasts such as comparing the two groups of subjects, then the test statistics should be adjusted. This method can be applied where the assumption of exchangeability between conditions or groups is possible e.g. one-sample t-test, paired t-test, two-sample t-test, ANOVA or other nonparametric equivalents such as rank sum.

Simulations

Sub-ROI time series extraction

In order to extract the time series for connectivity analysis, we first averaged the data across vertices to generate the mean sub-ROI time course. To avoid signal cancellation, the averaging took into account the polarity mismatches that occur because of MNE estimate spreading across sources whose orientations were not aligned. This was done by flipping the polarity of the signals from sources that were oriented at greater than 90° relative to a principal direction of the cortical normals within the sub-ROI. This process results in 2D matrix, which are epochs by time.

Time Frequency decomposition

The 2D epoched time series was then convolved with a dictionary of complex Morlet wavelets (each spanning seven cycles), resulting in 3 dimensional complex spectra epoch-time-frequency matrix ($S_k(t,f)$).

Coherence computation

The coherence between each sub-ROI pair (from ROI_1 - ROI_2) was computed using normalized cross-spectrum, mathematically:

$$C_{xy}(t,f) = \left\| \frac{\sum_{k=1}^{n} Sx_k(t,f) \,.\, {Sy_k}^*(t,f)}{\sqrt{\sum_{k=1}^{n} |Sx_k(t,f)|^2}\sqrt{\sum_{k=1}^{n} |Sy_k(t,f)|^2}} \right\|$$

Where * is the complex conjugate. The magnitude of cross-coherence varies between 0 and 1, a value of 0 again indicating a complete absence of synchronization at the given frequency f in the time window centered on t, and 1 indicating perfect synchronization. The frequency range for the simulation data were from 5-50 Hz and for the real dataset from 7-65 Hz.

Normalized Coherence

In the real data set example, we eliminated the statistical bias due to the non-Gaussian distribution of coherence values and unequal sample sizes, as well as the problem of spurious coherence [1], by using Z-Coherence [2], a normalized coherence measure in which the principal condition (Deviant, a total of 50 trials per participant) is normalized with respect to a baseline condition (Standard, a total of 150 trials per participant). Z-Coherence is defined mathematically as:

$$Z - C_{xy}(t,f) = \frac{\left(tanh^{-1}\left(\left|C_{xy}^{a}(t,f)\right|\right) - \left(\frac{1}{N_1 - 2}\right)\right) - \left(tanh^{-1}\left(\left|C_{xy}^{b}(t,f)\right|\right) - \left(\frac{1}{N_2 - 2}\right)\right)}{\sqrt{\left(\frac{1}{N_1 - 2}\right) + \left(\frac{1}{N_2 - 2}\right)}}$$

Z-Coherence was used for real data set to eliminate the spurious coherence. We should note that PeSCAR is not dependent on the functional connectivity estimate. Any method such as imaginary coherence or phase locking value can be used.

Comparison with existing methods

In order to see the statistical power of PeSCAR, we compared it with conventional averaging.

Conventional Averaging

Time series were averaged across the ROI separately for left and right STG for each subject and condition. Coherence was estimated between left-right STG. Cluster statistics in time and frequency were used to find the difference between two conditions.

Varying sub-ROI size

Here, we tested whether varying the sub-ROI size has any influence on the statistical results. Using freesurfer, left and right STG were divided into 5, 7, 11 and 13 sub-ROIs respectively. We repeated the simulation using the scaling factor of 0.05, resulting in a relatively lower SNR. By dividing the STG into smaller number of sub-ROIs (5, or 7), the surface for each sub-ROI will be larger than dividing the STG into 9 sub-ROIs. In contrast, dividing the STG into 11 and 13 sub-ROIs, the surface will be smaller than having 9 sub-ROIs in STG. Consequently; the number of sub-ROIs where the signal was generated was adjusted slightly according to the STG division. Therefore, the signal generated in STG was within 1, 2, 3 and 4 sub-ROIs when dividing the STG into 5, 7, 11 and 13 sub-ROIs respectively. Note that when we had 9 sub-ROIs for STG, the signal was generated within the first 3 sub-ROIs. In

Figure S1 the results are demonstrated. Basically, there is no difference if we divide the ROI in various equal size. The effect between the conditions is still recoverable.

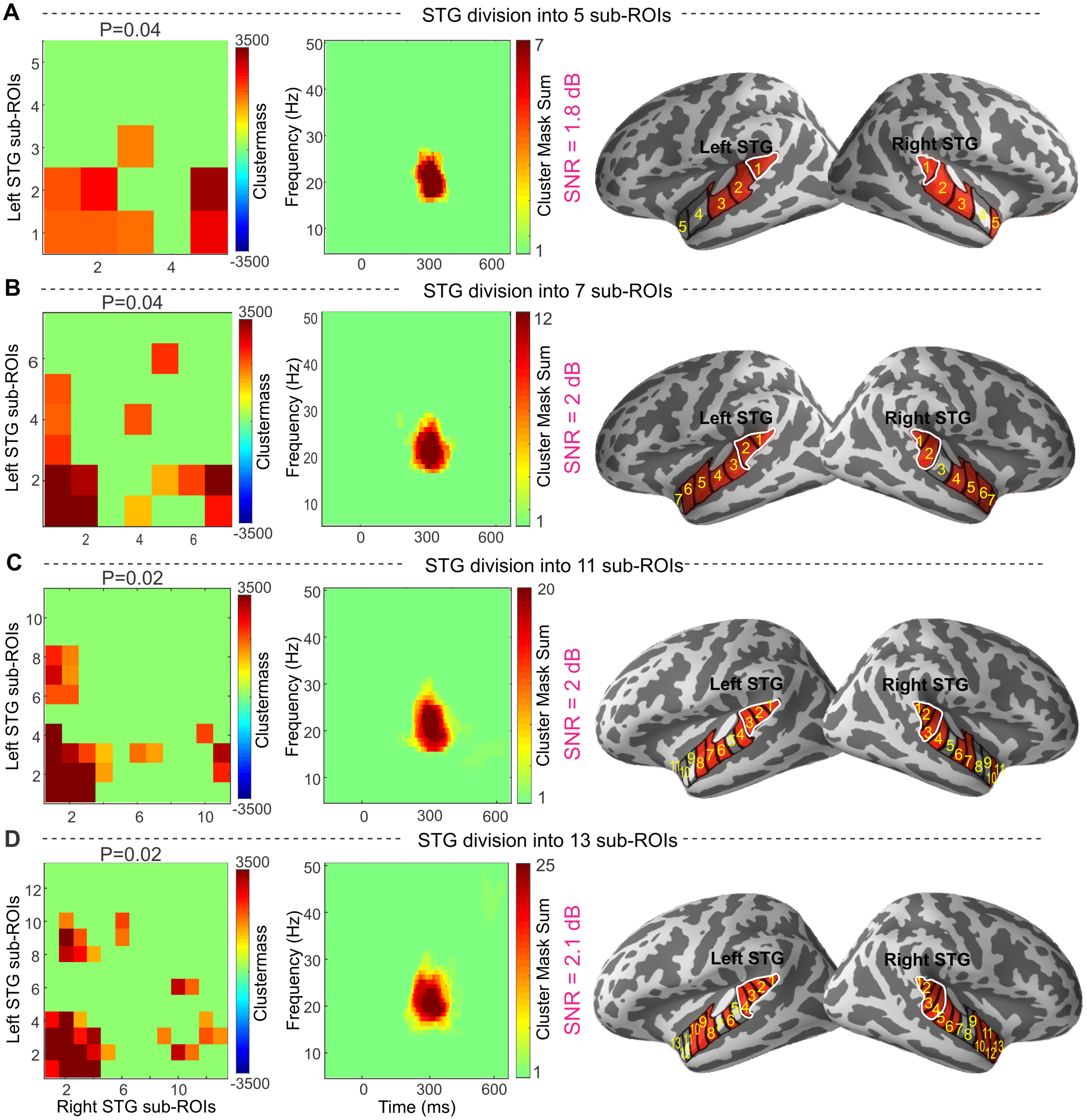

Figure S1: *Left-right STG functional connectivity comparison between two simulated conditions (scaling factor of 0.05, SNR of 2 dB for 9 sub-ROI case) where STG is divided into 5 (**A**), 7 (**B**), 11 (**C**) and 13 (**D**) sub-ROIs. The actual simulated signal was placed in the sub-ROIs with white color border (right column). In each panel, left column (**A-D**) is the original total connectivity matrix demonstrating sub-ROI pairs that reached significance; middle column (**A-D**) is the un-weighted time frequency map of the difference between conditions and right column (**A-D**) is the sub-ROIs on the cortex.*

Human data example: Data collection and preprocessing

Seventeen healthy volunteers with an average age of 12 (±2) participated in this study. Subjects completed a passive auditory task, so called mismatch negativity (Nataanen, et al, 1993). The experiment consisted of two conditions: standard and deviant. Standard stimulus was a complex tone consisted of ten sinusoidal tones starting at 500 Hz with 25% frequency increments between them. The deviant mismatch stimulus was a complex tone constructed by raising the frequency of each tone in the standard by 30%. Deviants occurred among standards with a relative frequency of 0.2 in a pseudorandomized fashion with the constraint that two deviants were separated by at least 3 standards and at most 7 standards. MEG data were acquired using a whole-head MEG system (Elekta-Neuromag), comprised of 306 sensors. In addition, all subjects underwent an anatomical scan using a 3.0 T magnetic resonance imaging (MRI) scanner (Siemens Medical Systems, Erlangen, Germany). MEG data were spatially filtered using the signal space separation method [4,5]. Cardiac and ocular artefacts were removed by signal space projection [6]. The data were filtered between 0.1 and 140 Hz. The data were epoched into single trials, from 400 ms prior to stimulus onset to 800 ms after it. Epochs were rejected if the peak-to-peak amplitude during the epoch exceeded 1000 fT/cm and 3000 fT in any of the gradiometer and magnetometer channels, respectively. The geometry of each participant's cortical surface was reconstructed from the 3D structural MRI data using FreeSurfer software (http://surfer.nmr.mgh.harvard.edu). The MEG forward solution was computed using a single-compartment boundary-element model (BEM) assuming the shape of the intracranial space [7]. The cortical current distribution was estimated using minimum-norm estimate (MNE) software (http://www.martinos.org/martinos/userInfo/data/ sofMNE.php) and assuming the orientation of the source to be fixed perpendicular to the cortical mesh. Each participant's inflated cortical surface was registered to an average cortical representation (FsAverage in FreeSurfer) by optimally aligning individual sulcal-gyral patterns computed in freesurfer [8]. For further details, please see [9]. We selected ROIs, which have been consistently found as the neural generators of mismatch negativity [10-12], namely, superior temporal gyrus (STG) and inferior frontal gyrus (IFG) consisted of parsopercularis, parstriangularis and parsorbitalis according to Freesurfer parcellation [13]. Each ROI was sub-divided into smaller sub-ROIs using an automatic routine provided in Freesurfer. All sub-ROIs in both ROIs were of approximately the same size. In total we ended up 9 temporal and 9 frontal sub-ROIs. Note that, in coherence analysis, larger sub-ROIs can lead to signal cancellation due to the sulcus geometry, which in turns decreases the signal to noise ratio [14].

Application to source activation

Let's assume there is one group of participants and they have done an experiment with two conditions (C_1 and C_2). The goal is to find differences in the ROI source activity between the C_1 and C_2. The processing steps are as

following and illustrated in Figure S2:

i. Divide the ROI into approximately equal size sub-ROIs.

ii. Calculate statistics within each sub-ROI between C_1 and C_2 using paired t-test. This will yield the original t-values sum.

iii. Employ permutation method to correct for multiple comparisons in space, i.e. over sub-ROIs:

a) Permute C_1 and C_2

b) Repeat step (ii) using the permuted conditions

c) Generate the null distribution based on the t-values from (b)

iv. Assign a single p-value to the ROI by comparing the original t-values from step (ii) with the null distribution.

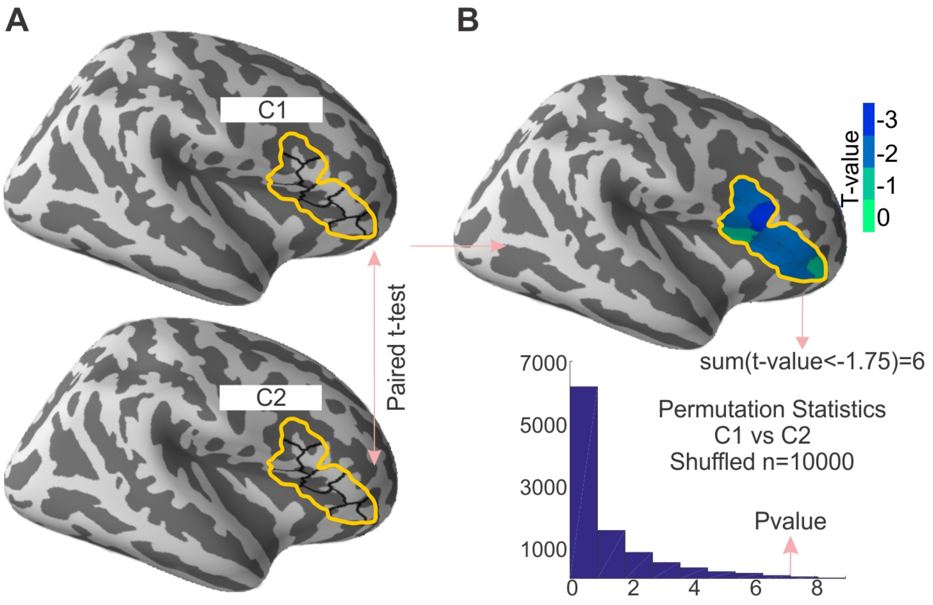

Figure S2: *Application of permutation statistics in regions of interest to compare source space activation. (**A**)* C_1 *and* C_2 *are compared using paired t-test for each sub-ROI within IFG. (**B**) T-values for each sub-ROI is mapped in right-top panel and a schematic of a null distribution in lower panel.*

Application to inter-measure correlations

Let's assume there is one group of participants and they have done an experiment. The goal is to find a correlation between ROI source activity and a behavioral measurement across the subjects. The processing steps are as following and illustrated in Figure S3:

i. Divide the ROI into approximately equal size sub-ROIs.

ii. Calculate the correlation between each sub-ROI and the behavioral measurement. This will yield original r-values.

iii. Employ permutation method to correct for multiple comparisons in space, i.e. over sub-ROIs:

a) Permute the behavioral scores across the subjects.

b) Repeat step (ii) using the permuted data.

c) Generate a null distribution using the r-values from (b).

iv. Assign a single p-value to the ROI by comparing the original r-values with the null distribution.

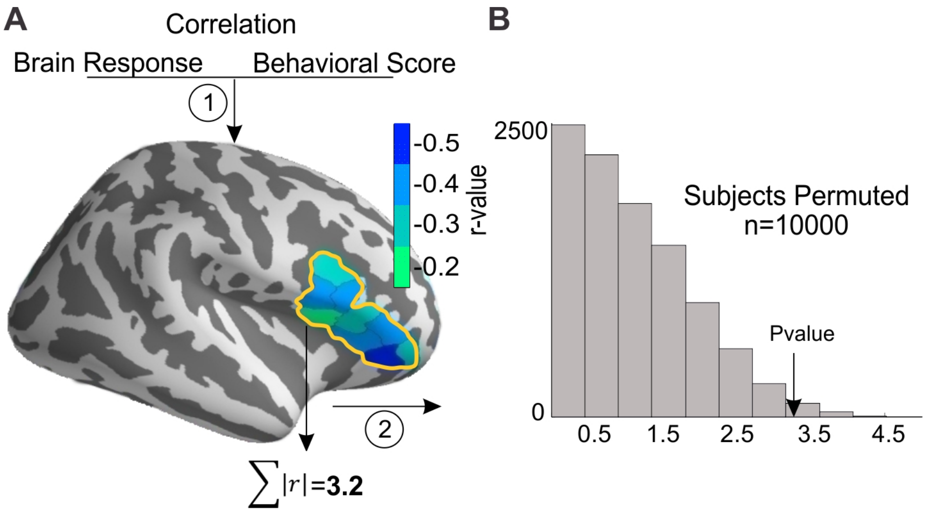

Figure S3: *Application of permutation statistics in regions of interest for inter-measure correlation. (**A**) Brain response is correlated for each sub-ROI with the behavioral score. R-values from the correlation analysis are mapped for each sub-ROI. (**B**) Histogram generated using* $\sum|r|$ *values from each permutation.*

References

1 Sekihara, K., Owen, J. P., Trisno, S. & Nagarajan, S. S. Removal of spurious coherence in MEG source-space coherence analysis. *IEEE transactions on bio-medical engineering* **58**, 3121-3129, doi:10.1109/TBME.2011.2162514 (2011).

2 Maris, E., Schoffelen, J. M. & Fries, P. Nonparametric statistical testing of coherence differences. *Journal of neuroscience methods* **163**, 161-175, doi:10.1016/j.jneumeth.2007.02.011 (2007).

3 Khan, S. *et al.* Local and long-range functional connectivity is reduced in concert in autism spectrum disorders. *Proceedings of the National Academy of Sciences* **110**, 3107-3112 (2013).

4 Taulu, S., Kajola, M. & Simola, J. Suppression of interference and artifacts by the Signal Space Separation Method. *Brain topography* **16**, 269-275 (2004).

5 Taulu, S. & Simola, J. Spatiotemporal signal space separation method for rejecting nearby interference in MEG measurements. *Phys Med Biol* **51**, 1759-1768, doi:10.1088/0031-9155/51/7/008 (2006).

6 Gramfort, A. *et al.* MNE software for processing MEG and EEG data. *Neuroimage* **86**, 446-460, doi:10.1016/j.neuroimage.2013.10.027 (2014).

7 Hämäläinen, M. S. & Sarvas, J. Feasibility of the homogeneous head model in the interpretation of neuromagnetic fields. *Phys Med Biol* **32**, 91-97 (1987).

8 Fischl, B., Sereno, M. I. & Dale, A. M. Cortical surface-based analysis. II: Inflation, flattening, and a surface-based coordinate system. *Neuroimage* **9**, 195-207 (1999).

9 Mamashli, F. *et al.* Auditory processing in noise is associated with complex patterns of disrupted functional connectivity in autism spectrum disorder. *Autism Res* **10**, 631-647, doi:10.1002/aur.1714 (2017).

10 Rinne, T., Degerman, A. & Alho, K. Superior temporal and inferior frontal cortices are activated by infrequent sound duration decrements: an fMRI study. *Neuroimage* **26**, 66-72, doi:10.1016/j.neuroimage.2005.01.017 (2005).

11 Naatanen, R., Paavilainen, P., Rinne, T. & Alho, K. The mismatch negativity (MMN) in basic research of central auditory processing: a review. *Clin Neurophysiol* **118**, 2544-2590, doi:10.1016/j.clinph.2007.04.026 (2007).

12 Garrido, M. I., Kilner, J. M., Stephan, K. E. & Friston, K. J. The mismatch negativity: a review of underlying mechanisms. *Clin Neurophysiol* **120**, 453-463, doi:10.1016/j.clinph.2008.11.029 (2009).

13 Destrieux, C., Fischl, B., Dale, A. & Halgren, E. Automatic parcellation of human cortical gyri and sulci using standard anatomical nomenclature. *NeuroImage* **53**, 1-15, doi:10.1016/j.neuroimage.2010.06.010 (2010).

14 Ahlfors, S. P. *et al.* Cancellation of EEG and MEG signals generated by extended and distributed sources. *Hum Brain Mapp* **31**, 140-149, doi:10.1002/hbm.20851 (2010).
